# Exploring the Economic Aspects of Hospitals: A Comprehensive Examination of Relevant Factors

**DOI:** 10.7759/cureus.54867

**Published:** 2024-02-25

**Authors:** Madhur Pradhan, Kiran T Waghmare, Rahma Alghabshi, Fathiya Almahdouri, Khalid M Al Sawafi, Iman M, Atka M Alhadhramy, Einas R AlYaqoubi

**Affiliations:** 1 Obstetrics and Gynaecology, Khoula Hospital, Muscat, OMN; 2 Management Studies, Mumbai University, Mumbai, IND; 3 Hospital Administration, Ibra Hospital, Ibra, OMN

**Keywords:** electronic health records, resources and infrastructure, solar energy, cost effectiveness analysis, hospital economy

## Abstract

Financial limitations in the hospital industry have the potential to exacerbate healthcare disparities, impede investments in cutting-edge medical treatments, as well as impair patient outcomes. The interdependent connection between a hospital economy and the general well-being of the community highlights the necessity of careful financial oversight and inventive healthcare policies. Effective collaboration among policymakers, healthcare administrators, and stakeholders is imperative in the development of sustainable economic models that give equal weight to fiscal prudence and optimal patient outcomes. This article aims to underscore the pivotal importance of strategic fund allocation guided by hospital administrators, accentuating several key initiatives capable of revolutionizing healthcare delivery and elevating the institution's stature within the medical community. The other important aspects discussed here are fund allocation in hospitals, the boom of online consultations, and emphasis on the use of sustainable and cost-effective modalities of energy.

## Introduction and background

This article aims to describe the economic factors influencing hospital operations, focusing on identifying strategies for sustainable financial management and improved patient care outcomes. Also, the paper presents insights to healthcare administrators, policymakers, and stakeholders on achieving a balance between cost-efficiency and the delivery of quality healthcare. In the realm of healthcare economics, the deliberate allocation of funds by hospital administrators assumes a paramount role in shaping both patient care quality and the overall efficiency of medical institutions. While investments in new medical technologies, diagnostic facilities, and clinic expansions remain essential, administrators must equally acknowledge the significance of allocating resources to other critical domains that profoundly influence medical excellence and foster superior patient outcomes.

From construction to infrastructure to staff management, pharmacy, supplies, use of alternative/green energy sources like solar energy, rainwater harvesting for water supply, decreasing the overall expenditure and increasing the revenues, hence enabling them to provide better facilities. Activities such as training, teaching, and research take a good number of finances, time, and various other resources of the institute. All these points have been elaborated on in detail and some of them are just briefly explained because of a lack of evidence and research conducted about them related to the hospital economy. K.J. Arrow, in 1963, emphasized a lot about the hospital economy. During those days, most of the physicians were working in either government hospitals or small clinics at home, even home visits by the doctors were very common. From those times till today, the health sector has grown so much and the economy has also grown to a level where healthcare economics forms a part of the overall economy of any country [1].

Health-care economics affects not just private and corporate hospitals, but the country and the state as a whole. The hospital economy is a dynamic and complex domain that necessitates a delicate balance between providing optimal patient care and sustaining the necessary infrastructure [2,3]. Hospital administrators face the critical task of managing expenses while ensuring the highest standard of healthcare delivery [4,5]. For this article, we have searched various databases like PubMed, Google Scholar, and Scopus using relevant keywords like hospital economy, healthcare, economic performance, healthcare expenditure, hospital expenditure, hospital charges, and hospital costs.

## Review

Cost management in healthcare

There are several aspects of cost management in healthcare. It involves medical/surgical supplies, equipment, labor, repairs, salaries, and incentives. All these expenses are important and cannot be compromised and at the same time, patient safety and care take priority over everything. Utilizing resources is made easier by using clinical guidelines and evidence-based approaches [6]. Advanced medical gadgets, telemedicine programs, and electronic health records are some examples of technology integration. Hospitals depend on multiple revenue streams outside of standard patient billing to ensure financial sustainability. Insurance reimbursements, government funding, philanthropy and donations, research and clinical trial revenue from grants from the pharmaceutical industry, strategic infrastructure investments, facility expansion, and the purchase of cutting-edge medical equipment are a few notable sources of income [7]. The hospital economy is a complex field that requires responsible financial management and effective patient care. By making well-informed decisions that optimize resources, maximize revenue, and ultimately assure the institution's long-term success in providing exceptional patient care, hospital administrators play a crucial role in determining the hospital economy [8].

Usually, any private hospital comprises infrastructure, machines, facilities, pharmacy, food and storage, manpower, research, training human resources, medical insurance, medical indemnity, marketing, etc. [9,10]. Hospitals that plan their infrastructure, keeping in mind the present needs and future expansion based on the market dynamics have better cost management as compared to hospitals that start without such planning [11]. Any unplanned changes done later, not only increase the cost substantially but also can lead to a loss of revenue (due to the temporary closure of the facility) [12,13]. For example, if hospital management decides on opening a facility for a cardiac catheterization laboratory (cath lab), that was not a part of the expansion / modified plan, it may need changes in infrastructure, shifting of some wards to other areas, temporarily closing that wing/floor/area for some time, etc and all that ultimately result in the revenue loss for that period. While adding the cathlab the overall inflow of cash will increase will increase in the long run.

In the dynamic realm of healthcare, strategic allocation of funds by hospital administrators plays a pivotal role in shaping the quality of patient care and the overall efficiency of medical institutions. While the acquisition of new gadgets, setting up diagnostic areas, and expanding clinics are crucial investments, administrators must also recognize the significance of allocating funds for other vital areas that can drive medical excellence and foster optimal patient outcomes. Fund allocation for patient-centric initiatives can significantly enhance patient satisfaction and overall healthcare experience. Administrators should prioritize investments in patient communication tools, patient engagement platforms, and programs that promote patient education. Patient-centric initiatives empower individuals to actively participate in their healthcare decisions, leading to better treatment adherence and improved health outcomes. Furthermore, allocating funds for patient feedback mechanisms enables hospitals to continuously improve their services and tailor patient care to meet specific needs.

Therefore, it is better to take into consideration the planning and modifications which will be needed in the future. This will help the hospitals minimize the cost of such expansions. Also, an “integrated service model” can be adopted to solve these problems. Integrated services/group practice is a very nice idea to have needed facilities in a very cost-effective way. For example, sharing an in vitro fertilization (IVF) lab by a group of gynecologists, to keep the maintenance cost low. However, sometimes the physicians feel a little diluted or neglected in others' place. Also, even patients may feel that they are not treated holistically or a personal touch is missing. These are some pros and cons of this approach [14].

Machines/facilities

There is always a debate about investing in certain facilities which can be shared with some other hospitals. For example, a computed tomography (CT) machine, magnetic resonance imaging (MRI), or a blood bank, having these facilities in-house definitely makes the hospital a one-stop arrangement for everything, but these facilities can be shared or outsourced by sending the patient to nearby center for MRI or having a blood storage unit rather than blood bank and keeping the cost at a minimum and yet not compromising on treatment. Whether it is high-end technology like infrastructure for robotic surgeries, intensive care unit comprising ventilators and other ICU equipment, modern anesthesia workstations, a state-of-the-art laboratory, monitors for an ambulance, point-of-care ultrasonography machines, all require the best possible strategies for allocation of the budget which actually is the most difficult factor to control against manpower and resources management and ultimately brings the art and science of hospital economy together. The whole essence of the hospital economy is very difficult to balance against the odds mentioned earlier in this section. Another example is the in vitro fertilization (IVF) lab, as discussed above, from establishment to maintenance the cost incurred to set up the facility is huge. Also, specialized manpower such as infertility specialists, embryologists, and nursing staff will be an added cost, which cannot be ignored. The patient inflow for such treatment won't be very high initially. Therefore, sharing and pooling of such resources is a better idea for having the facility and keeping the cost to a minimum [15].

Pharmacy

Procurement of medicines is a major activity in any hospital. difference between government and private hospitals is that even a basic drug tendering process will be followed the most competitively priced gets the business and the same drug is available for all the patients. Whereas, in private setups, the drug that fetches the maximum margin will be preferred by the management. This increases the cost for the patients. So even if it seems a very profitable activity for the hospital, we are not sure if the hospital will lose out on such patients in the future who may go to other cheaper options. Such activities might also lead to a loss of reputation and hence will affect the revenue in the long term [16]. Pharmacy plays an important role in healthcare economics and is responsible for patient outcomes, cost management, and the overall efficiency of healthcare delivery [17]. The rationale use of medications based on evidence and local guidelines improves patient health and reduces readmissions and complications, thus providing better patient satisfaction [18].

Electronic health record (EHR) system

EHR sounds to be an expensive investment, but in the long run, it is cost-effective and a very efficient system for keeping records. Also, there is less wastage of paper and hence it is environmentally friendly as well [19]. It has been observed that patients are poor in keeping records of past medical treatment and hence the EHR system helps big time in keeping records. Hospitals cannot function without EHR, which is essential in streamlining patient care, clinical decision-making, and record-keeping. EHR facilitates billing procedures, improves coding accuracy, and supports data-driven decision-making. Thus, EHR leads to streamlining billing, and record-keeping and thus helps in financial sustainability [20].

Health insurance

The availability of health insurance has increased access to many facilities and more patients are getting the benefits of the same. The dynamics of the hospital economy are significantly shaped by health insurance, which has an impact on financial results, the provision of patient care, and resource allocation. Clinicians and hospital executives alike must comprehend the relationship between health insurance and its effects on hospitals because it is a crucial part of the healthcare system. As it is a large source of funding for medical services, health insurance has a big impact on hospital revenue streams. Hospitals' reimbursement for care is influenced by insurance reimbursement models including fee-for-service and value-based care. Access to healthcare services is boosted when patients have health insurance, which boosts patient volumes and diversifies the patient base for hospitals. The financial viability of hospitals is significantly impacted by the availability of health insurance and the timely clearance of insurance money from the companies. Higher percentages of insured patients frequently result in hospitals being more financially stable because they are consistently paid for their services. The key to establishing financial sustainability is balancing the patient mix and diversifying revenue streams through multiple insurance contracts and alliances [21].

Use of natural resources

Electricity consumption in any hospital is huge due to the 24x7 usage of air conditioners, various machines, boilers, etc. Hence solar energy can be a very cheap option for hospitals. Many parts of the world are blessed with good sunlight most time of the year. In Middle Eastern countries, the temperatures are soaring hot almost throughout the year. Temperatures are in high 40 degrees centigrade in the summer and even in winter in the range of 20 degrees centigrade. To make use of this enormous free natural energy, the initial cost might be higher but in the long run, it’s one of the cheapest energy sources available on Earth [22]. Hospitals have a lot of requirements for both potable and non-potable water. For non-potable usage of water, rainwater harvesting is one of the methods being utilized in many parts of the world. In this system, the rainwater is collected from rooftops and other places and diverted to the system, where it is filtered and reutilized for various non-potable purposes such as gardening, flushing toilets, etc. the hospitals [23]. Healthcare organizations spend a lot of money on energy consumption. Solar energy is becoming more popular among administrators to save costs [22]. Hospitals can generate electricity on-site, reducing their dependency on traditional energy sources and electricity costs, by investing in solar panels and renewable energy infrastructure [24,25]. Key benefits of solar energy adoption are reduced operational expenses, favorable return on investment, and a positive environmental impact.

Water recycling

Many hospitals face the challenge of managing water resources efficiently [26]. Water recycling is the only alternative to handle this situation. Water recycling systems provide hospitals with an additional water source during periods of water scarcity or disruptions in the municipal water supply [27]. This enhances the hospital's resilience and ability to sustain operations during challenging times [28]

Various other green sources of electricity generation such as wind turbines, geothermic energy, hydro electric-power etc. which are easily available to the hospitals should be explored as they will reduce the electricity cost a lot. These alternative or green sources of energy are the most cost-effective solution not only for hospitals but also for the overall nation [29]. Another solution is not to have a centralized air conditioner system, because most of these units are on throughout even if the facility is unused. Not only air conditioners but other electrical fixtures like tube lights, bulbs, fans, geysers, etc. should be handled and managed effectively to lower the overall electrical consumption. These small but very effective changes might look negligible but, infusing these practices into the system will make each one of us a more disciplined and concerned person on planet Earth [30].

Catering services in hospitals

In hospitals that serve packaged plate food to patients/attendants, it has been observed that despite having a very interesting food menu more than half of the food gets wasted. Either the served portions are large or not of the patients/attendants' preferred choices as it is impossible to serve everyone’s individual food preferences it’s better to have a section-wise centralized food system where, out of the available food options, everyone gets to choose what and how much they want to eat hence reducing the wastage to a great extent. In many developing countries and various government hospitals, the use of a food trolley is still preferred where everything is served whatever is needed and hence will lead to a reduction in wastage [31,32]. Some researchers think that hospitals should be more involved in the food industry to reduce or prevent the impact of diseases, quality control the food packed/prepared, and by reducing readmissions, by changing the food served to patients [33].

Pandemic lessons

The COVID-19 pandemic time taught us many lessons. Suddenly everything became manageable at home just by patient education [34]. Management of patients at home by maintaining social distancing and following basic hygiene. It not only worked well overall but also led to less staffing in hospitals, a smaller number of patients and their relatives, research, and the development of vaccines. We not only learned various important things conquering the pandemic COVID-19 but also learned some important lessons for the future also. The COVID-19 pandemic brought some major changes in administrative affairs which were not only cost-effective but were patient-friendly and prevented hospital visits and waiting lists, especially for outpatient departments [35]. Many other lessons, not directly related to the hospital economy are like online teaching and work from home which picked up during the pandemic and are continued with the same or maybe better results. In online teaching/working from home, people are in the comfort of their homes, spending less time in traffic and spending more time with their families. Megacities had better air quality index due to less pollution.

Online consultations revolutionized healthcare delivery

As the pandemic necessitated physical distancing and limited in-person interactions, hospital administrators swiftly embraced telemedicine solutions to ensure continuous patient care. Online consultations emerged as a transformative alternative, enabling patients to receive medical advice and treatment remotely [36]. Physicians and specialists leveraged secure video conferencing platforms to conduct consultations, diagnose conditions, and prescribe medications. This cost-effective approach not only reduced the burden on healthcare facilities but also ensured uninterrupted care for patients, especially those with chronic conditions who required regular follow-ups. Online consultations facilitated seamless care, ensuring that patients could access medical advice and prescriptions from the safety of their homes, mitigating the risk of infection. Telemedicine extended medical services to remote or underserved areas, improving healthcare access and reducing geographic barriers for patients seeking specialized care. Patients were spared the need to travel to healthcare facilities, resulting in time and cost savings for both patients and healthcare providers [37,38].

Online Continuing Medical Education (CME) activities

The pandemic underscored the need for healthcare professionals to stay updated on rapidly evolving medical knowledge. Hospital administrators responded by rapidly transitioning to online CME activities, allowing healthcare professionals to access accredited educational programs virtually [39]. Webinars, online conferences, and interactive modules became a staple in healthcare education, empowering clinicians to learn about the latest research, evidence-based practices, and emerging treatment modalities. Key benefits of Online CME Activities are flexibility and convenience, cost-effective learning, global collaboration, having online medical courses, and eventually upskilling the healthcare workforce [40].

Funds allocation

Fund allocation for patient-centric initiatives can significantly enhance patient satisfaction and overall healthcare experience. Administrators should prioritize investments in patient communication tools, patient engagement platforms, and programs that promote patient education. Patient-centric initiatives empower individuals to actively participate in their healthcare decisions, leading to better treatment adherence and improved health outcomes [11]. Furthermore, allocating funds for patient feedback mechanisms enables hospitals to continuously improve their services and tailor patient care to meet specific needs.

Hospital administrators should recognize the importance of investing in their most valuable asset-their healthcare workforce. Allocating funds for staff development, training, and wellness programs fosters a positive and supportive work environment. Such initiatives improve employee satisfaction, reduce burnout, and enhance staff retention. A content and motivated healthcare workforce is more likely to provide compassionate and efficient patient care, contributing to an overall positive hospital reputation and patient outcomes [41].

Enhanced recovery after surgery (ERAS) pathways

ERAS pathways are another example of a cost-effective model, especially for surgical patients. These pathways comprise patient education, pre-op control of comorbidities, use of regional anesthesia and multimodal analgesia techniques, optimum pain relief, etc. A combination of all this results in early discharges, and hence fewer days of hospitalization, better patient satisfaction, and finally having more bed turnover thus improving revenue generation for the hospital [42]. In the pursuit of delivering patient-centric care and optimizing healthcare resources, hospital administrators are increasingly turning their focus to innovative strategies that yield improved patient outcomes while reducing the burden of in-hospital stays and healthcare costs. Enhanced Recovery After Surgery (ERAS) pathways have emerged as a game-changing initiative in perioperative care, revolutionizing the patient journey from preoperative preparation to postoperative recovery [43].

Some policies like a good appraisal of deserving candidates, retaining good staff, training the lacking but potential candidates, and if needed sacking the non-performing or undeserving candidates who are not showing any growth and improvement in their work, should improve hospital efficiency leading to increased output and revenues [11]. But at the same time, understaffing or non-skilled workers may lead to more medical negligence.

Nowadays medical negligence cases and their associated cost on physicians and health institutions and practices like getting indemnity/insurance can give peace of mind and at the same time smooth functioning at work.

Cost of litigations

Clinical error and malpractice claims are two facets of medical practice that are becoming increasingly important [44,45]. There is a worry that the possibility of contracting a disabling illness as a result of medical intervention while a patient is hospitalized is increasing the cost of care, adding to the patient’s burden, and increasing the costs for the healthcare system and society as a whole due to malpractice claims [46,47].

The increase in the number of hospitals has made the hospital business very competitive. This has led to increased marketing costs. To increase the number of patients in small and medium-level hospitals some malpractices such as incentives for referring doctors are on the rise. To maintain the quality, various countries have their respective health standards like Joint Commission International (JCI) accreditation [18]. However, getting these accreditations is a very expensive and time-consuming procedure. All these indirect costs are eventually recovered from the patients which eventually leads to higher healthcare costs.

Integrating Artificial Intelligence ‎(AI)-driven health information quality into hospital economic strategies

AI's ability to process and interpret vast amounts of health data revolutionizes hospital ‎budgeting and financial planning. This new wave of technological integration brings with it ‎a promise of unprecedented efficiency and accuracy in managing healthcare resources. By ‎leveraging AIs' analytical prowess, hospitals can now predict patient inflows, optimize ‎staffing, and manage inventory with a previously unattainable precision. This leads to cost ‎savings and enhances the overall quality of patient care.‎ Furthermore, AI-driven systems are instrumental in identifying trends in patient care, ‎enabling hospitals to allocate funds more effectively to areas that require the most attention. ‎This targeted allocation of resources ensures that investments are made that directly benefit ‎patient outcomes, improving healthcare services.‎ However, this integration is not without its challenges. Standardizing AI across different ‎healthcare settings requires a careful balance between technological advancement and the practicalities of healthcare economics. Hospitals must navigate the complexities of ‎implementing these systems - from initial costs to staff training and data security.‎ Moreover, the ethical implications of AI in healthcare cannot be overlooked. As we entrust ‎more of our decision-making processes to algorithms, the need for transparency and ‎accountability in AI systems becomes paramount. Ensuring that these systems are efficient ‎but also fair and unbiased is crucial [48]‎.

## Conclusions

In conclusion, the complex dynamics of the hospital economy have a significant impact on healthcare providers as well as the larger society. Hospitals' financial health has an impact well beyond their walls as they strike a careful balance between providing high-quality patient care and remaining financially viable. Strong hospital economies support medical innovation, hire trained and committed medical staff, and guarantee access to state-of-the-art equipment-all of which improve the quality of care provided by hospitals as a whole. Sustainable energy use and effective fund allocation should be taken seriously by investors and administrators. Online consultations are here to stay and will be utilized by patients and clinicians in the future more effectively. 
